# Coalescent inference for infectious disease: meta-analysis of hepatitis C

**DOI:** 10.1098/rstb.2012.0314

**Published:** 2013-03-19

**Authors:** Bethany Dearlove, Daniel J. Wilson

**Affiliations:** 1 Nuffield Department of Clinical Medicine, Experimental Medicine Division, University of Oxford, Oxford, UK; 2 Wellcome Trust Centre for Human Genetics, University of Oxford, Oxford, UK

**Keywords:** coalescent, epidemiology, hepatitis C, metapopulation, SIR

## Abstract

Genetic analysis of pathogen genomes is a powerful approach to investigating the population dynamics and epidemic history of infectious diseases. However, the theoretical underpinnings of the most widely used, coalescent methods have been questioned, casting doubt on their interpretation. The aim of this study is to develop robust population genetic inference for compartmental models in epidemiology. Using a general approach based on the theory of metapopulations, we derive coalescent models under susceptible–infectious (SI), susceptible–infectious–susceptible (SIS) and susceptible–infectious–recovered (SIR) dynamics. We show that exponential and logistic growth models are equivalent to SI and SIS models, respectively, when co-infection is negligible. Implementing SI, SIS and SIR models in BEAST, we conduct a meta-analysis of hepatitis C epidemics, and show that we can directly estimate the basic reproductive number (*R*_0_) and prevalence under SIR dynamics. We find that differences in genetic diversity between epidemics can be explained by differences in underlying epidemiology (age of the epidemic and local population density) and viral subtype. Model comparison reveals SIR dynamics in three globally restricted epidemics, but most are better fit by the simpler SI dynamics. In summary, metapopulation models provide a general and practical framework for integrating epidemiology and population genetics for the purposes of joint inference.

## 1. Introduction

During an ongoing outbreak, understanding the epidemiological dynamics and predicting the likely course of the outbreak are time-critical tasks essential for informing intervention [1,2]. If systematic monitoring is in place, key parameters such as *R*_0_, the basic reproductive number [1], can be estimated directly, as in the case of the foot and mouth disease outbreak among British cattle in 2001 [3] and the outbreaks of severe acute respiratory syndrome in Asia in 2002 and 2003 [4]. Genetic analysis provides a window into the epidemic history of a pathogen that can complement epidemiological analysis, as in the case of the H1N1 influenza A pandemic in 2009 [5,6], or take its place in the absence of reliable surveillance data. The ability to sequence pathogen genomes in real time, for example during the 2010 cholera outbreak in Haiti [7], foretells of the increasingly important role for genetic analysis during outbreak response.

Genetic analysis is a well-established tool for revealing the epidemic history of pathogen populations [8,9]. It commonly involves the post hoc interpretation of an evolutionary tree constructed from genetic sequences. Relationships between isolates may reveal the order of transmission events [10,11], whereas the shape of the tree is informative about overarching dynamics [12]. However, more powerful approaches explicitly integrate genetic and epidemiological models. For example, coalescent methods—which can be used to infer historical changes in population size [13–15]—have been applied to pathogen populations to infer historical changes in prevalence. By modelling changes in prevalence using the susceptible–infectious–susceptible (SIS) model, epidemiological parameters such as the intrinsic growth rate of the epidemic have been estimated directly [16].

Early applications of the coalescent approach shed new light on the epidemic behaviour of the hepatitis C virus (HCV) [16], and the pathogen has continued to attract intense research attention owing to its medical importance and amenability to genetic analysis. HCV is a major cause of liver disease, including cirrhosis and liver cancer. Estimated to infect 160 million people around the world [17], it is implicated in 350 000 deaths per year [18]. Sharing contaminated needles and transfusion of infected blood products are thought to be the main routes of transmission [19]. HCV is an enormously diverse RNA virus, comprising six major types with varying geographical distributions [20,21]. Coalescent inference has been used to date the origin of HCV in different countries [16,22–28], providing a historical context for the emergence of epidemics and providing quantitative support for the roles of iatrogenic transmission [22] and drug use [29].

The advent of population-level whole genome sequencing has revealed previously unfathomed diversity in pathogenic bacteria [30], leading to wider interest in integrated approaches to genetics and epidemiology beyond rapidly evolving viruses such as HCV. However, theoretical work has shown that although the central assumption of coalescent approaches—that effective population size is proportional to prevalence—is valid at dynamic equilibrium [31], it does not hold more generally [32,33]. In this study, we derive a new framework for population genetic inference of epidemiological dynamics based on a metapopulation model of pathogen populations. Using coalescent results for metapopulations [34,35], we expose the assumptions implicit to coalescent approaches and explore the limits of genetic inference. We implement SI, SIS and SIR models in BEAST [36], and conduct a meta-analysis investigating the epidemiological processes that underlie differences in genetic diversity between HCV epidemics.

## 2. Models

### (a) Metapopulation model of pathogen populations

**Figure 1.**
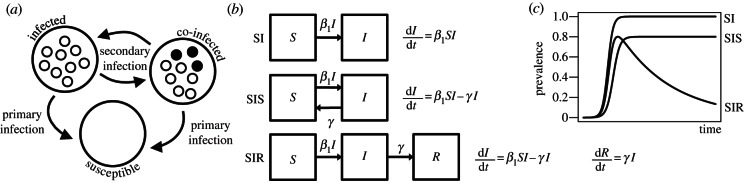
Metapopulations and epidemiological dynamics. (*a*) Pathogen populations are metapopulations because they exist as an aggregate of isolated subpopulations within individual hosts. We refer to infection of susceptible hosts as primary infection, and subsequent infection events as secondary infection. We use compartmental models from epidemiology to model the dynamics of the metapopulation. (*b*) The SI, SIS and SIR models are simple compartmental models. Changes in the proportions of susceptible (*S*), infected (*I*) and recovered (*R*) hosts are modelled using differential equations. In all three models, the proportion of infected hosts is assumed to increase at rate **β**_1_*SI*, where **β**_1_ is the primary transmission coefficient. In the SIS model, hosts clear infection and return to the susceptible class at rate **γ**. In the SIR model, hosts that clear infection recover and are no longer susceptible. (*c*) The models predict different epidemiological dynamics. In the SI model, the whole population is eventually infected. In the SIS model, a dynamic equilibrium is reached. In the SIR model, the epidemic peaks and burns out as the supply of susceptible hosts is exhausted.

Metapopulations (literally populations of populations [37,38]) have been used to account for heterogeneity in pathogen species caused by strain structure or host structure [39,40]. However, pathogen populations are metapopulations in a more fundamental sense, because the population is an aggregate of the many isolated subpopulations colonizing individual hosts (figure 1).

The key feature of a metapopulation that distinguishes it from other structured populations is the extinction of individual demes (i.e. subpopulations) and their re-colonization by other demes [41]. In pathogens, demes correspond to hosts, colonization corresponds to infection of an uninfected host (what we call primary infection) and extinction corresponds to clearance of infection. Migration to a colonized deme corresponds to secondary infection of an infected host. To make a concrete population genetics model, additional assumptions are required [34,35,41], principally that (i) upon primary infection the infecting genotypes come from a single host, and (ii) the carrying capacity is immediately attained within the newly infected host.

Among the advantages of using the metapopulation model is the wealth of understanding of metapopulation dynamics [37,38,41–43]. In a series of papers, Wakeley [44–46] developed coalescent approximations for structured populations, including metapopulations [34,35], based on the assumption that the number of colonized demes is large. The main result from his work is that under disparate, complex models of population structure, the genealogy of individuals sampled from different demes is well approximated by a standard coalescent process whose effective population size is a function of the demographic parameters. This puts inference for metapopulations on a practical footing [36], and the assumption that the number of infected hosts is large is consistent with the deterministic compartmental models commonly used in epidemiology.

### (b) Compartmental models of infectious disease

Compartmental models are important tools for modelling infectious disease dynamics [1]. In a simple SI model, the proportions of all hosts that are susceptible (*S*) and infectious (*I*) are modelled using differential equations. Usually, the total rate of primary infection is assumed to depend on the number of susceptible and infectious individuals and a transmission coefficient (**β**_1_). This is known as strong proportionate mixing [1]. In the SIS model, infected individuals clear infection and return to the susceptible class at rate **γ**. In the SIR model, individuals that recover from infection instead become immune. These three models have different dynamics, with the SIR model producing the classical epidemic expansion and burn out (figure 1).

Initially, when infection is rare and susceptible hosts are plentiful, the epidemic increases exponentially with rate *r*_0_, the intrinsic growth rate. In the SI model, *r*_0_ = **β**_1_ and in the SIS and SIR models, *r*_0_ = **β**_1_ − **γ**. During this exponential phase, the transmission rate per infection is **β**_1_, but it slows as susceptible hosts are exhausted. The clearance rate **γ** corresponds to the inverse of the average duration of infection. An important quantity is the basic reproductive number *R*_0_, defined as the total number of infections caused by an index case in a totally susceptible population [1]. In the SIS and SIR models, *R*_0_ = **β**_1_*/γ*. In the SIS model, *R*_0_ determines the equilibrium prevalence, whereas it determines the peak prevalence in the SIR model.

Compartmental models can be elaborated endlessly. However, the only extension to the basic models we make is to consider the dynamics of secondary infection. Assuming strong proportionate mixing, it follows that the total rate of secondary infection depends on the square of the number of infectious individuals and a transmission coefficient (**β**_2_). Although this is important for the metapopulation model, our treatment of secondary infection does not change the dynamics of the epidemiological models. As noted, the use of deterministic differential equations to model epidemic dynamics implies the number of infected hosts is large. Although this cannot hold in the early stages of the epidemic, experience suggests these models are nevertheless useful for epidemiological inference [3–5].

## 3. Results

### (a) Effective population size

The key parameter in a coalescent model is *N*_e_, the effective population size, because it determines the coalescence rate, which in turn determines relatedness within the sample [15]. In the metapopulation model described earlier, the many-demes limit [34,35] gives the effective population size as3.1
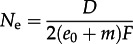


where
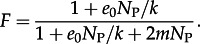


In these equations, *D* is the number of infected hosts, *e*_0_ is the rate of primary transmission per infection, *m* is the rate of secondary transmission per infection, *N*_P_ is the pathogen population size within a host and *k* is the number of genotypes transmitted during primary infection. *F* is the inbreeding coefficient, which is the probability that two individuals sampled within the same host are descended from the same transmission event. See table S1 in the electronic supplementary material for all parameter definitions.

Assuming strong proportionate mixing, the rates of primary and secondary transmission per infection are *e*_0_ = **β**_1_*S* and *m* = **β**_2_*I*, respectively, which yields3.2
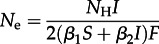


where
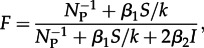


and where *N*_H_ is the total number of hosts. Equations (3.1) and (3.2) resolve the apparently conflicting observations that (i) *N*_e_ is proportional to prevalence at dynamic equilibrium [31], but (ii) changes in prevalence do not necessarily induce a linear change in *N*_e_ [33] because the rates of primary and secondary transmission per infection and the inbreeding coefficient depend, in general, on prevalence. This is true under assumptions of both strong and weak proportionate mixing. For further explanation of the determinants of effective population size in the metapopulation, see electronic supplementary material, figure S1.

### (b) Coalescent SI and SIS models

Equations (3.1) and (3.2) are consistent with the results of a simpler model [33], which assumes co-infection is negligible (**β**_2_ = 0). Because this assumption will often be reasonable, and because it reduces the number of parameters to be estimated, we embrace it in the rest of what follows. The SI and SIS models can be solved in closed form (see §5 and equations (5.1) and (5.2)), so it is possible to write down the effective population size under these models. For the SI model, the effective population size simplifies to3.3
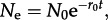


which is an exponential growth curve with parameters *N*_0_ = *N*_H_(1−*S*_0_)*/*(2**β**_1_*S*_0_), the effective population size at present, and *r*_0_, the intrinsic growth rate. Time is measured from the present (*t* = 0) back into the past (*t* > 0). For the SIS model, the effective population size simplifies to3.4
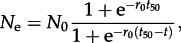


which is a logistic growth curve with parameters *N*_0_, *r*_0_ and *t*_50_ = −log(*r*_0_/(**γ**(1−*S*_0_))−1)/*r*_0_, the time at which *N*_e_ reached half its maximum.

Equations (3.3) and (3.4) show that the exponential and logistic growth curves, which are commonly used in coalescent analyses of pathogen effective population size [14,23,29], arise from simple SI and SIS models under the assumptions of strong proportionate mixing and no co-infection. However, the growth curves describing changes in *N*_e_ are simpler than the underlying growth curves that describe changes in prevalence, and have one fewer parameter. Consequently, there is no one-to-one correspondence between the coalescent parameters and the epidemiological parameters, meaning that the epidemiological parameters cannot be fully identified from genetic analysis alone. An independent estimate of one of the epidemiological parameters (e.g. rate of clearance of infection or present-day prevalence) is required to reconstruct historical changes in prevalence. In this respect, our results differ from Pybus *et al.* [16], but we agree with their key result that the intrinsic growth rate (*r*_0_) in an SIS model can be estimated by modelling changes in *N*_e_ using a logistic growth curve. We also agree that to estimate the basic reproductive number *R*_0_, an independent estimate of one of the epidemiological parameters is needed.

### (c) Coalescent SIR model

Equations for the epidemiological dynamics in the SIR model cannot be solved analytically, but can be solved numerically using computational techniques [47]. Unlike the simpler models, there is no confounding of epidemiological parameters, meaning that, in principle, all the parameters of the epidemiological model (see table S1, electronic supplementary material) can be estimated from genetic data alone. Consequently, *R*_0_ can also be estimated, in principle, directly from genetic data. We found that model comparison and parameter estimation using BEAST were aided by the following re-parameterization: *N*_0_ = *N*_H_(1 − *S*_0_ + **γ**log(*S*_0_)*/(*β**_1_)/(2**β**_1_*S*_0_), the effective population size at present, *r*_0_ = **β**_1_ − **γ**, the intrinsic growth rate, **γ**, the rate of clearance and *t*_peak_, the time since the epidemic peaked, which must be calculated numerically.

### (d) Meta-analysis of hepatitis C

To investigate the practical value of our approach for estimating epidemiological parameters, reconstructing epidemic history and explaining variation in genetic diversity between epidemics, we conducted a meta-analysis of HCV, one of the most intensively studied pathogens in the context of joint evolutionary–epidemiological inference. We conducted a literature search for HCV datasets with well-described sampling frames and readily available metadata. Initially, we identified 28 datasets for which subtype, sampling location, prevalence and *NS5B* gene sequences were available [22,23,25,29,48–59]. However, we excluded those with small sample size (fewer than 20 sequences) and evidence of recombination (see the electronic supplementary material, table S2). Recombination is problematic for coalescent inference [60] and provides evidence of co-infection, which our method assumes is absent. In total, 18 datasets satisfied our incorporation criteria (see the electronic supplementary material, dataset S1).

**Figure 2.**
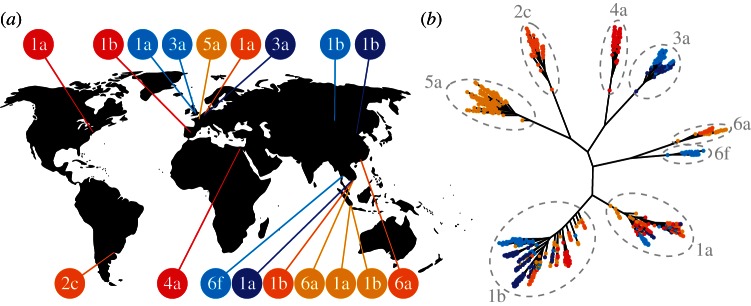
Summary of hepatitis C datasets. (*a*) The geographical distribution of HCV datasets analysed, with country of origin and subtype indicated. Colours differentiate datasets of the same subtype. (*b*) Maximum-likelihood genealogy of all sequences based on a global alignment of the *NS5B* gene. Subtypes are indicated with dashed lines. Sequences are colour-coded as in (*a*) to distinguish datasets of the same subtype from different countries. A square-root transformation was applied to branch lengths to aid visualization.

Figure 2 shows the geographical distribution of the HCV datasets and a genealogy based on a global alignment of all sequences, with the subtypes indicated. Subtypes formed distinct monophyletic groups, but the ancestral histories of datasets within the same subtype were shared to varying degrees. We fitted our coalescent SI, SIS and SIR models to each dataset separately while bearing in mind this overlap. For the meta-analysis, we estimated *N*_0_ (the effective population size at the time of sampling) and *r*_0_ (the intrinsic growth rate) using a model-averaging approach that assumed equal prior probability of each scenario (SI, SIS and SIR).

**Figure 3.**
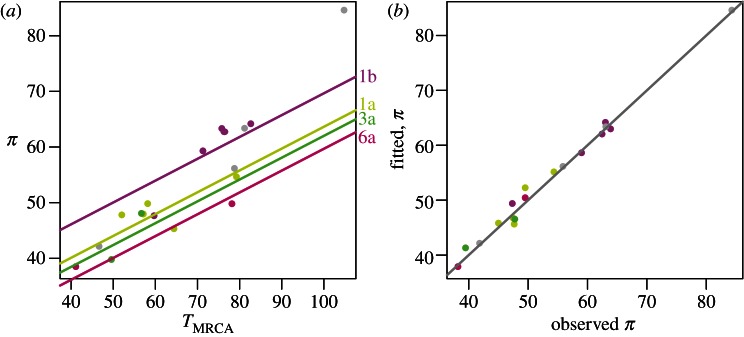
Meta-analysis of HCV diversity. Results of the regression of genetic diversity (**π**) against age of the most recent common ancestor (*T*_MRCA_), subtype, intrinsic growth rate (*r*_0_) and population density. Intrinsic growth rate was not significantly associated with **π** after accounting for the other effects. (*a*) Scatterplot of **π** against *T*_MRCA_, with regression lines shown for subtypes represented by multiple datasets. (*b*) Fitted diversity against observed diversity. The *R*^2^ for the regression was 98.9%.

We used linear regression to explore the epidemiological determinants of genetic diversity between epidemics. We measured genetic diversity using **π**, the mean number of nucleotide differences between HCV sequences in the same dataset. Diversity varied considerably, ranging from **π** = 20.3 to **π** = 84.3 per kilobase (see the electronic supplementary material, table S2). We found that the strongest predictor of diversity was the age of the most recent common ancestor (*T*_MRCA_), followed by population density and subtype (figure 3). Table 1 shows the regression coefficients and *p*-values, although the latter must be viewed with a degree of caution owing to pseudo-replication within subtypes. The overall predictive power of the regression was very high (*R*^2^ = 98.9%). Epidemics with older *T*_MRCA_ had substantially higher diversity as would be expected, whereas increased population density predicted a reduction in diversity. Of the subtypes represented by multiple datasets, 1b had highest diversity and 6a had lowest diversity after correcting for the effects of *T*_MRCA_ and population density. Surprisingly, there was no significant relationship between diversity and intrinsic growth rate, *r*_0_, after taking into account other factors. This would be explained by rapid epidemic growth across the datasets, resulting in star-shaped genealogies.

**Table 1.** Linear regression of HCV diversity.

model: **π** = *T*_MRCA_ + *r*_0_ + subtype + population density				
coefficients				
	estimate	s.e.	*F*-test	*p*-value
intercept	25.3	5.93		
*T*_MRCA_	0.456	0.0719	40.2	0.0004
pop. density	−0.0287	0.00638	20.2	0.0028
subtype	n.a.	n.a.	6.48	0.0124
*r*_0_	6.373	11.7	0.297	0.6027
multiple *R*^2^ = 98.9%				
subtypes				
	estimate	s.e.	*t*-test	*p*-value
1b versus 1a	3.04	1.48	2.06	0.0782
2c versus 1a	0.222	2.73	0.081	0.9374
3a versus 1a	0.981	2.07	0.473	0.6507
4a versus 1a	12.6	3.68	3.41	0.0112
5a versus 1a	−3.05	2.43	−1.26	0.2495
6a versus 1a	−4.33	2.01	−2.15	0.0682
6f versus 1a	−2.85	2.34	−1.22	0.2631

**Figure 4.**
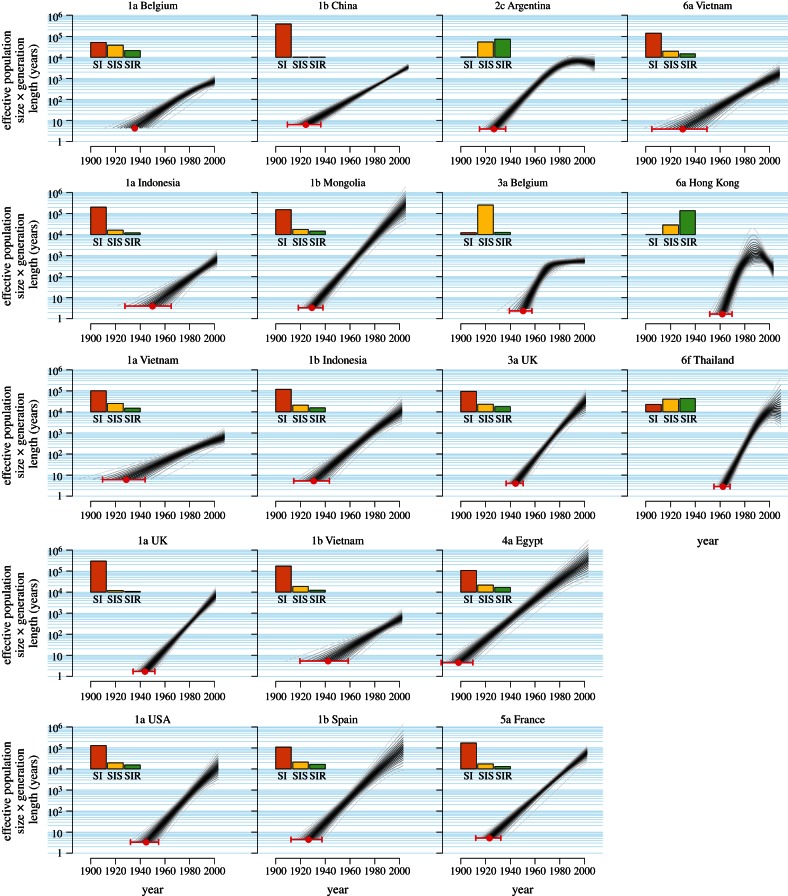
Reconstructed effective population size with model averaging. For each of the 18 datasets, the reconstructed effective population size is plotted against time. The datasets are labelled by HCV subtype and sampling location. For each dataset, the grey lines show the quantiles, at 5% increments, of the posterior distribution of *N*_e_, averaged over models (endemic, SI, SIS and SIR). Quantiles closer to the median are shaded darker. The results for all datasets are plotted on the same log_10_ scale. The time of the MRCA is indicated with a red circle (posterior median) and error bar (95% credible interval). Inset for each dataset, the posterior probability (PP) of the underlying epidemiological model (SI, SIS or SIR) is shown as a bar chart. The PP for the endemic model is not shown because it was less than 0.2% for every dataset.

Reconstructing historical changes in *N*_e_ revealed that most datasets exhibited strong exponential growth, consistent with the SI model (figure 4). For each dataset, we calculated the posterior probability (PP) of the SI, SIS and SIR models, and a model of endemic infection that implies a constant effective population size (see the electronic supplementary material, table S3). The endemic model was rejected outright for every dataset (PP ≤ 0.002). In 13 cases, the SI model was clearly preferred (PP = 0.62–0.99). In the subtype 1a dataset from Belgium, SI dynamics were most probable (PP = 0.44), but there was also support for the SIS (PP = 0.36) and SIR models (PP = 0.20). Only in one example—subtype 3a in Belgium—was the SIS model most probable (PP = 0.88). The preference for the simpler SI dynamics in most of the datasets is evidence that these epidemics have neither reached dynamic equilibrium, as in the SIS model, nor begun to burn out, as in the SIR model. All the epidemics except one (subtype 4a in Egypt) appear to have emerged during the past 100 years, reiterating the important role of twentieth century phenomena such as blood transfusions and needle sharing in the global spread of HCV [22,29].

### (e) Examples of SIR dynamics in hepatitis C

In three datasets, the SIR model was preferred over the others: subtype 2c in Argentina, 6a in Hong Kong and 6f in Thailand. Only in the case of the SIR model can all the epidemiological parameters be estimated directly from genetic data alone. Consequently, we were able to estimate *R*_0_ and reconstruct historical changes in prevalence for these three epidemics. Because the total number of hosts is a parameter, we were able to obtain separate estimates for prevalence (as a proportion) and the total number of infected hosts.

**Figure 5.**
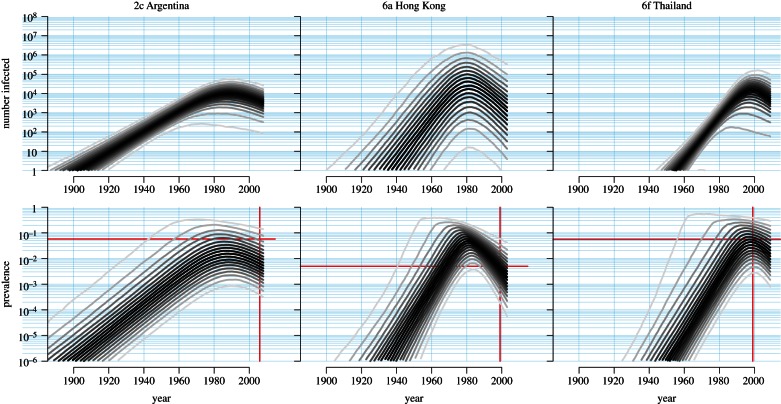
Reconstructed SIR dynamics: numbers of infected hosts and prevalence. For subtypes 2c in Argentina, 6a in Hong Kong and 6f in Thailand, the SIR model was preferred to the SI or SIS models. When the data supports the SIR model, changes in the number of infected hosts and prevalence can be inferred directly from genetic data. These are shown for each of the three datasets. The grey lines show the quantiles, at 5% increments, of the posterior distribution. Quantiles closer to the median are shaded darker. In the prevalence plot for each dataset, the intersection of the red lines indicates the independent estimate of point prevalence [56,58].

HCV-2c is generally uncommon but in the Córdoba province of Argentina it is the dominant subtype, found in 50 per cent of cases or more [54,58]. From 1880 to 1920, the central regions of Argentina, of which Córdoba is part, received an influx of European migration, mainly from Italy where subtype 2c is also common [54]. The PP of SIR dynamics in HCV-2c in Córdoba was 53.8 per cent, with the SIS model next most likely (PP = 45.4%). We reconstructed historical changes in the number of infected individuals and prevalence under the SIR model (figure 5). The *T*_MRCA_ was dated to between 1915 and 1936. Initially, the epidemic grew exponentially with a doubling time (log(2)/*r*_0_) between 3.6 and 6.7 years (see the electronic supplementary materials, table S3). We estimated that the epidemic peaked some time between 1969 and 2002 and has fallen since.

Subtype 6a is common in Hong Kong, accounting for 23.6 per cent of all HCV infections and 58.5 per cent of HCV infections in intravenous drug users [61]. It is a relatively recent epidemic [55]. The rarity of HCV-6a in China led to the suggestion that HCV-6a was introduced from Vietnam, where it is dominant, during peaks of immigration around 1979 and 1992 [61]. SIR dynamics were most probable in this dataset (PP = 71.0%), but there was also some support for the SIS model (PP = 28.7%). We dated the *T*_MRCA_ to between 1952 and 1962, following which the number of infections grew rapidly with a doubling time between 0.7 and 3.8 years. We estimated that the number of HCV-6a infections in Hong Kong peaked in 1986, with a broad 95 per cent credible interval of 1963–1993.

The many subtypes of HCV type 6 are distributed throughout Asia, but HCV-6f appears to be restricted to Thailand, where it is the most common form (56%) [48]. Our analysis revealed marginally greater support for the SIR model over the SIS model (PP = 39.6% versus 38.1%). The difficulty discriminating between the two scenarios is a consequence of very recent deceleration in the spread of the epidemic. We dated the *T*_MRCA_ to between 1955 and 1968. Using the SIR model, we reconstructed the historical number of infected hosts and prevalence (figure 5). We estimated a doubling time between 1.4 and 3.6 years, and dated the peak prevalence to between 1964 and 2008.

Although the HCV-6 epidemics in Hong Kong and Thailand appeared to have faster intrinsic growth rates than the HCV-2c epidemic in Argentina, we obtained similar estimates for *R*_0_ and average duration of infectivity for all three datasets. We estimated basic reproductive numbers of 1.20 (95% CI 1.04–5.51), 1.44 (1.08–13.2) and 1.42 (1.07–19.0) in Argentina, Hong Kong and Thailand, respectively. We estimated average durations for the infectious period (1/**γ**) of 1.47 years (95% CI 0.27–27.0), 1.24 years (0.26–14.1) and 1.55 years (0.28–40.0), respectively. We compared the reconstructed prevalence in the three epidemics with contemporary estimates of point prevalence in the three sampling locations [56,58]. These estimates are indicated in figure 5 by the intersection of the red lines. In all three cases, prevalence estimated by independent epidemiological investigation fell within the 95 per cent credible interval of prevalence reconstructed from genetic data.

## 4. Discussion

Using a metapopulation model of pathogen populations, we have developed a new approach for integrated genetic and epidemiological inference. We derived a formula for the effective population size in a pathogen population that reconciles previous results [8,31,33] and provides rationale for widely used genetic analyses. Specifically, we showed that using exponential and logistic growth curves to analyse historical changes in pathogen effective population size is equivalent to assuming underlying SI and SIS dynamics when co-infection is absent.

Using BEAST to implement our models, we conducted a meta-analysis of 18 HCV datasets from across the world. As expected, we found the age of the MRCA to be the strongest predictor of the diversity of an epidemic. Surprisingly however, there was no relationship between intrinsic growth rate and diversity after accounting for age of the MRCA, population density and subtype. This observation is consistent with rapid growth during the exponential phase of the epidemics. Under rapid growth, the MRCA is only marginally younger than the epidemic. Therefore, it follows that HCV diversity can be used as rough guide to the age of an epidemic.

We found evidence for SIR dynamics in three datasets: subtype 2c in Argentina, 6a in Hong Kong and 6f in Thailand. Using the coalescent SIR model, we were able to directly estimate the basic reproductive number and historical changes in prevalence and in the absolute number of infected hosts in these epidemics. We obtained similar estimates of *R*_0_ in the three epidemics (1.2–1.4), although there was substantial uncertainty. This value is considerably lower than previous estimates, largely because the duration of the infectious period that we estimated (1.2–1.6 years) was substantially shorter than the 10–30 years that have previously been supposed [16]. Estimating short infectious periods for hepatitis C is surprising in view of the nature of the disease, which is chronic in 80 per cent of people and has lifelong infectivity [17,18]. One possible interpretation could be that the majority of transmission occurs shortly after infection. However, the broad 95 per cent credible intervals were consistent with infectious periods up to 27, 14 and 40 years, respectively.

There may also be an element of ascertainment bias to this result because we can infer only SIR dynamics and *R*_0_ once an epidemic has passed its peak, which is likely to occur sooner when *R*_0_ is smaller. However, the three epidemics exhibiting SIR dynamics shared features in common other than *R*_0_. All three were globally rare but locally dominant subtypes. The Argentinean and Hong Kong epidemics appear to have been introduced originally by migration [54,61], while both the Hong Kong and Thai epidemics emerged relatively recently. Dynamical modelling shows that the number of infectious individuals falls when the number of susceptible individuals becomes exhausted. Why this should occur more quickly in these epidemics than the global subtype 1a and 1b outbreaks is unclear, but may depend on mode of transmission, the behaviour of risk groups, local competition between subtypes and virological differences.

Our approach has a number of assumptions and limitations, chief among which is the assumption that the number of infected hosts is large. Although this assumption is consistent with the use of deterministic compartmental models, it cannot possibly be true at the beginning of the epidemic. There are a number of promising avenues for incorporating stochasticity into combined genetic and epidemiological models. Particle Markov Chain Monte Carlo (MCMC) has been developed to fit stochastic, nonlinear dynamics to gene genealogies, although currently the genealogy is assumed to be known [62]. Branching processes have been used as an alternative to the coalescent; however, the approach is currently limited to simple birth–death processes [63,64]. Stochastic demography is readily incorporated into the coalescent [65], and this will be an area of further investigation.

Random mixing is a common assumption in compartmental models of epidemiological dynamics that is difficult to justify empirically. Theoretical work shows that variance in network connectivity substantially affects epidemiological dynamics and hence genetic diversity [31,66–68]. There is hope that such variability can be handled using a more general formulation of the metapopulation model than was needed here [34], in which different classes of hosts, such as super shedders, are explicitly modelled. Another of our assumptions, that co-infection is absent, is likely to prove more difficult to overcome. When there is co-infection, recombination can occur. We found evidence of recombination in some HCV datasets, which we excluded from further analysis. Although attempts have been made to incorporate recombination into population genetic inference [69], these methods are generally computationally prohibitive.

There are a number of other extensions to our approach that we have left for future research. Changes in the size of the host population are readily incorporated into our model, and this might prove fruitful for inference if independent data are available to disentangle the effects of host and pathogen population dynamics, for instance by coupling an analysis of host and pathogen genetic diversity in BEAST. When there is no more than a single pathogen sequence per host, as we assumed here, longitudinal sampling is straightforward to account for using the standard technique [70] as implemented in BEAST, with no adjustments necessary to the model. When there are multiple pathogen sequences per host, the genealogy of the metapopulation is conceptually divided into the *scattering* and *collecting* phases [34], which correspond informally to within- and between-host evolution, respectively. New apparatus would be required for inference in this situation.

For our analyses, we used a simple HKY85 substitution model [71], ignoring heterogeneity in the molecular clock rate between sites, codon positions and branches of the tree. However, detailed analyses suggest that such heterogeneity does occur in HCV [26,72]. One of the benefits of implementing our approach in BEAST is that this complexity can be readily incorporated in future analyses. There has been considerable variation in the estimates of the molecular clock rate in HCV [72]. We assumed a clock rate of 0.58 × 10^−3^ substitutions per site per year, which was estimated for the *NS5B* gene [73], and was previously applied to a number of the datasets we analysed. However, there is evidence to suggest that the rate may be closer to 1.0 × 10^−3^ per site per year [26,72]. The effect of underestimating the clock rate would be to systematically overestimate the dates of events during the epidemic history, while overlooking uncertainty and heterogeneity in the clock rate will cause the credible intervals for some of our parameters and dates to be anti-conservative.

One of the important points our work demonstrates is that there are limits to what may be inferred about epidemiological dynamics from genetic data. For example, 13 of the 18 datasets were best fit by the simplest, SI model. Although this model contains none of the biological complexity inherent to HCV epidemiology, on statistical grounds, there was no support for even modest elaborations of the SIS or SIR models. The SI, SIS and SIR models may be caricatures of true epidemiological dynamics, but they capture key features of epidemic processes, including exponential, plateau and burn-out phases. In this study, we directly compared the goodness-of-fit of endemic, SI, SIS and SIR models. In practice, a useful approach might be to include the non-parametric Bayesian skyline plot [74] in the model comparison [72]. This would allow rejection of the parametric models if none adequately described the population history of the sample. In such a case, the Bayesian skyline plot might help motivate and direct the construction of new, more realistic, parametric models via our metapopulation approach.

Another limitation of genetic inference, revealed by our theoretical results and in agreement with previous work [16], is that *R*_0_ cannot be directly estimated from genetic data in the coalescent SIS model because, although the intrinsic growth rate (*r*_0_) is well identified, the transmission coefficient (**β**_1_) and rate of loss of infection (**γ**) cannot be disentangled. In stochastic models, **β**_1_ and **γ** and therefore *R*_0_ can, in principle, be deconfounded, but if deterministic models are any guide, precise estimates cannot be expected unless additional information is available concerning, for example, the rate of clearance or prevalence. Fortunately, *r*_0_ will often be a convenient proxy for *R*_0_ because it exhibits the same threshold behaviour: when *r*_0_ ≥ 0 (equivalently, *R*_0_ ≥ 1), the infection persists in the population and when *r*_0_ < 0 (equivalently, *R*_0_ < 1), the epidemic dies out. The intrinsic growth rate is well identified from genetic data during the exponential growth period of the epidemic, in contrast to *R*_0_, which is not even well defined under the SI model.

Based on comparisons to independent estimates, the SIR model appeared to provide good predictions of prevalence (figure 5). However, we saw that only once an epidemic had peaked could the SIR model be fitted (figure 4). This has repercussions for the utility of genetic analysis for predicting an outbreak in real time. Although the intrinsic growth rate can be estimated during the exponential growth phase of the epidemic, it is not sufficient to predict the course of the epidemic. Independent estimates of quantities such as the duration of infection and point prevalence would be needed for prediction. Consequently, the role of genetic analysis in real-time prediction of outbreaks will be to complement, but not replace, epidemiological approaches.

The metapopulation analogy provides a firm grounding for combining population genetics and epidemiology. We have shown how it can be used to derive coalescent models with underlying SI, SIS and SIR dynamics that are readily used for practical analysis. With richer genetic data, it will become possible to detect microevolution on epidemiological timescales in many more pathogen species [30]. Joint genetic and epidemiological inference is a fertile area for research, and the machinery underlying our metapopulation approach [34] provides building blocks for arbitrary elaboration on the basic pattern we explored here.

## 5. Methods

### (a) Epidemiological and coalescent models

To obtain the effective population size for the metapopulation model, we adapted the results of Wakeley & Aliacar [34] and Wakeley [35] assuming haploidy and the propagule-pool model [41] for colonization (equation (3.1)). To model changes in metapopulation dynamics over time, we used simple SI, SIS and SIR compartmental models (figure 1). For parameter estimation, we made the simplifying assumption that co-infection is negligible. In the case of the SI and SIS models, we were able to obtain analytical solutions for the effective population size using the following closed-form solutions for the proportion of susceptible hosts, *S*, as a function of time. For the SI model,5.1
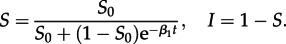
For the SIS model,5.2
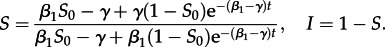


All parameter definitions are summarized in the electronic supplementary material, table S1. For the SIR model, a solution for *S* cannot be obtained analytically. However, assuming that the number of recovered individuals is initially zero gives the relationship5.3
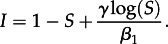


This simplifies the system of differential equations in the SIR model to a single ordinary differential equation that can be solved numerically:5.4
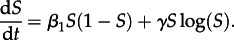


In the coalescent with demographic growth, the pairwise coalescence rate is the inverse of the effective population size, and calculation of the probability density of a genealogy under the coalescent model requires the calculation of the integrated coalescence rate [13]:5.5
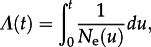


(elsewhere we suppress the dependency on time to avoid cluttered notation). Assuming no co-infection (**β**_2_ = 0), we can write this integral as a differential equation5.6
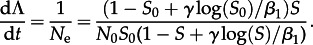


Because the effective population size is dependent on *S*, equations (5.4) and (5.6) define a system of differential equations to be solved together. We implemented this as an extension to BEAST [36] in Java using a fifth-order Cash–Karp Runge–Kutta method with adaptive stepsize control [47]. We also re-implemented the logistic growth function in BEAST because our parametrization for the SIS model uses *N*_0_, the effective population size at the present, rather than the carrying capacity. Example XML code and details of the Bayesian analysis are provided in the electronic supplementary material, text S1.

### (b) Meta-analysis

We searched the literature for HCV datasets with well-described sampling frames for which subtype, sampling location, prevalence and *NS5B* gene sequences were available. We initially identified 28 datasets, but we excluded a further 10 that had small sample size (fewer than 20 sequences), evidence of recombination or questionable sampling on further investigation. We used a simple permutation test based on the correlation between physical distance and three measures of linkage disequilibrium (*r*^2^, |*D*′| and G4), implemented as part of omegaMap [75]. We excluded a dataset if the null hypothesis of no recombination was rejected at the 5 per cent level by any of the three tests. This is not unduly conservative because of the similarity between the measures of linkage disequilibrium. Details of all 28 datasets are available in the electronic supplementary material, text S2. We performed multiple sequence alignment using the Geneious alignment tool [76] to produce a global alignment of all sequences and where an alignment was not available between sequences within the same dataset. All the alignments that we analysed are available in the electronic supplementary material, dataset S1.

For each of the 18 datasets that met our incorporation criteria, we calculated mean pairwise genetic diversity (**π**) and collated data on subtype, prevalence, host population size and population density (see the electronic supplementary material, text S2). We obtained point estimates of *T*_MRCA_, *N*_0_ and *r*_0_ averaged over models. We used multiple regression to explore the effect of these covariates on **π**. In the final model, we included all statistically significant covariates and *r*_0_, as we had strong prior interest in the inferred regression coefficient for this covariate.
